# Excessive DAO inhibits myoblast migration, leading to impaired myotube fusion and muscle strength decline by reducing ECM

**DOI:** 10.3389/fbioe.2025.1606357

**Published:** 2025-10-27

**Authors:** Xiang Liu, Jianchao Xue, Yiming Liang, Zhenggang Li, Rui Xu, Huaimei Yang, Yu Zhao, Qiyang Wang, Jianhong Hou, Sheng Lu

**Affiliations:** ^1^ Department of Orthopedics, The Key Laboratory of Digital Orthopaedics of Yunnan Province, The Clinical Medicine Center of Spinal and Spinal Cord Disorders of Yunnan Province, The First People’s Hospital of Yunnan Province & The Affiliated Hospital of Kunming University of Science and Technology, Kunming, China; ^2^ Department of Orthopaedic Surgery, Peking Union Medical College Hospital, Chinese Academy of Medical Sciences and Peking Union Medical College, Beijing, China; ^3^ Medical School, Kunming University of Science and Technology, Kunming, China

**Keywords:** AOC1, sarcopenia, DAO, Fbln1/FAK pathway, metabolic-biomechanical crosstalk

## Abstract

**Introduction:**

The molecular mechanisms underlying muscle strength decline, particularly in age-related conditions such as sarcopenia, remain poorly understood. Previous omics studies have suggested a potential association between AOC1 (encoding diamine oxidase, DAO, mainly expressed in intestine) and muscle weakness, but experimental validation and mechanistic insights are lacking. This study aims to investigate the influence of secretory DAO on muscle function and its potential receptors.

**Methods:**

Serum DAO levels were measured in elderly participants (n = 129) using ELISA and correlated with grip strength. Animal models included naturally aging C57 mice, rapidly aging SAMP8 mice, and a glycerol-induced acute muscle injury model were established to verify the role and content of DAO under overall condition. *In vitro* studies used C2C12 myoblasts treated with recombinant human DAO to assess migration, fusion, and cytotoxicity. Mechanistic insights were explored via mass spectrometry, co-immunoprecipitation, Western blotting, and immunofluorescence.

**Results:**

Elderly males with low grip strength exhibited significantly higher serum DAO levels, while no such correlation was observed in females. Aged and injured mouse models showed elevated DAO content in skeletal muscle, accompanied by fast-twitch fiber loss. In C2C12 cells, adding DAO recombinant protein inhibited myoblast migration and fusion without affecting viability. Mechanistically, DAO bound to Fbln1, suppressed FAK phosphorylation (Y576/Y577), and disrupted cytoskeletal remodeling.

**Discussion:**

This study provides experimental evidence that exssive DAO impairs muscle strength by inhibiting myoblast migration and fusion via the Fbln1/FAK pathway. The findings reveal a potential regulation between different organs and cells, and highlight a novel metabolic-biomechanical uncoupling mechanism linked by DAO in sarcopenia.

## 1 Introduction

Muscle strength is a cornerstone of the locomotor system, enabling essential functions such as posture maintenance, movement execution, and force generation (Li et al., 2024). Declines in muscle strength, as seen in sarcopenia or age-related dynapenia, directly impair mobility, increase fall risks, and exacerbate comorbidities like osteoporosis and metabolic disorders (Fogarty et al., 2019). The integrity of muscle force production relies not only on metabolic homeostasis but also on precise biomechanical coordination, where mechanosensitive proteins translate physical stimuli into biochemical signals to regulate muscle adaptation and repair (Tieland et al., 2018).

The generation of muscle strength about the coordinated action of actin and myosin filaments within muscle fibers (Latchman et al., 2023). The process begins with the sliding of these filaments past each other, creating tension and force. This is regulated by calcium ions and ATP, which provide the energy for muscle contraction. As the smallest unit of muscle fibers, recent advancements in muscle strength research have highlighted the importance of skeletal muscle cells and their predecessor, satellite cells (SCs). SCs are activated by stimuli such as exercise and injury, or secretory factors from other organs like liver and intestine, leading to their proliferation and differentiation into myoblasts (Bonaldo and Sandri, 2013). These myoblasts fuse with existing muscle fibers, contributing to muscle growth and repair. Except the structure factors, the strength and duration of muscle contractions are also influenced by neural signals, hormonal levels, and the overall state of the body and important metabolic organs (Wiedmer et al., 2021).

To explore the factors affecting muscle strength, particularly age-related and injury-induced muscle decline, researchers utilize population-based sequencing technologies. Among those results, many genome-wide association studies (GWAS) have identified SNPs in AOC1 linked to muscle strength (Jones et al., 2021), though its exact role here is unclear. AOC1 encodes diamine oxidase (DAO), an enzyme crucial for breaking down histamine and other polyamines (Neumann et al., 2012). AOC1 variants are associated with reduced DAO activity, leading to histamine accumulation and contributing to conditions like fibromyalgia, ADHD, and insomnia (Kirschner et al., 2014; Ding et al., 2022; Okutan et al., 2023). Despite these findings in various diseases, the specific function of AOC1 in skeletal muscle remains to be elucidated.

When conducting one study on the pathogenesis of osteoporosis and sarcopenia in our hospital, serum DAO levels were also measured in individuals with normal and reduced muscle strength. We found that men with muscle weakness had significantly higher circulation DAO level than those with normal muscle strength, but this was not observed in women. To further explore the link between serum DAO levels and muscle strength, we established three animal models: C57 mice with naturally aging-related muscle weakness, rapidly aging transgenic mice, and glycerol-induced acute injury models. The DAO level in skeletal muscles can be detected higher in low grip and injury group, which remains us there maybe is receptor for gathering DAO. *In vitro*, adding recombinant human DAO protein cultured mouse C2C12 cells inhibited myoblast migration and fusion. To uncover why overdose DAO disrupts myoblast behavior, we used mass spectrometry to identify receptors in skeletal muscle cells for DAO. Most of the candidates related to the cytoskeleton, cell adhesion, and extracellular matrix. Co-IP and Western blotting suggested DAO likely binds to and degrades Fbln1, impairing mechanical signaling. This study offers new insights into mechanical signaling regulation in myoblasts and proposes a novel dialogue mechanism between metabolism and mechanotransduction.

## 2 Materials and methods

### 2.1 Human serum DAO ELISA

Serum samples were collected from participants after obtaining informed consent in the Orthopedics Department of the First People’s Hospital of Yunnan Province (ethics code KHLL2024-YJ040) from November 2024 till December. Inclusion and Exclusion criteria: Over 60 years old, without neurological complications, or trauma, or disability. DAO levels in serum were measured using a commercial ELISA kit (#DL-DAO-Hu, Develop, Wuxi, China) according to the manufacturer’s instructions. Absorbance was read at 450 nm, and concentrations were calculated using a standard curve.

### 2.2 Grip strength test in mice

C57BL/6J male mice were housed under standardized conditions (12 h light/dark cycle, 22 °C ± 1 °C, 50%–60% humidity) with *ad libitum* access to food and water. All procedures were approved by the Institutional Animal Care and Use Committee of KUST (SYXK-K2023-0013). 3 in 4M and 3 in 18M mice were obtained. Combined four-limb grip strength were assessed using a digital grip strength meter (Model SA415, Lab Anim Tech, Jiangsu, China). Mice were acclimated to the testing environment for 30 min prior to trials. For each measurement, the mouse was positioned on a wire mesh platform. The platform was inverted, and the mouse was gently pulled downward until release. Peak force was recorded. Each mouse underwent three consecutive trials per session, with 5-min intervals between trials. The highest value from three trials was used for analysis. Tests were performed at consistent times in 4 days to minimize circadian variability.

### 2.3 Glycerol-induced muscle injury model

To establish an acute muscle injury model, 4-month-old male C57BL/6J mice were anesthetized with isoflurane and divided in two groups (6 for each). A total volume of 0.05 mL of 50% (v/v) glycerol in phosphate-buffered saline (PBS) was injected into the posterior compartment of the right hind limb to induce muscle injury. The contralateral left hind limb received an injection of an equal volume of PBS alone, serving as an internal control. All injections were performed percutaneously using a 29-gauge insulin syringe. Mice were euthanized 3 days post-injection. The gastrocnemius (GA) muscles from both injured (glycerol-injected) and control (PBS-injected) limbs were carefully dissected. In each group, 3 mices muscle was snap-frozen in liquid nitrogen for subsequent RNA extraction, while another 3 was fixed in 4% paraformaldehyde for histological analysis.

### 2.4 Histological analysis and immunofluorescence of mouse muscle tissue

Muscle tissues were fixed in 4% paraformaldehyde, embedded in paraffin, and sectioned at 5 μm thickness. Immunofluorescence: Sections were stained with antibodies against MYH1 (Anti-Fast Myosin Skeletal Heavy chain Rabbit pAb, GB112130, Servicebio, Wuhan, China) and MYH7 (Anti-Slow Skeletal Myosin Heavy chain Mouse mAb, GB121857, Servicebio, Wuhan, China), followed by appropriate secondary antibodies. Nuclei were counterstained with DAPI (P0131, Beyotime, Shanghai, China).

### 2.5 *In Vitro* cell culture and treatment

C2C12 myoblasts were obtained from Cas9X bio company, cultured in growth medium (DMEM with 10% FBS and 1% penicillin/streptomycin) at 37 °C and 5% CO2. Cells were treated with gradient concentrations (0, 100, 200, 500 pg/mL) of recombinant human AOC1 protein (#HY-P7497, MCE, Shanghai, China) for 24 h∼3 days. When testing fusion, cells were cultured around 90% confluent, then started treatment with differentiation medium (DM: DMEM supplemented with 2% horse serum (HS, Invitrogen) and 1% penicillin/streptomycin) to induce myogenic differentiation. When clear myotubes in control group can be observed under microscope, cells were fixed, permeabilized, and stained with antibodies against myosin heavy chain (MHC) (#ab37484, abcam, 1:2000) and DAPI to visualize nuclei. The cell viability was assessed using the Cell Counting Kit-8 (CCK-8, Dojindo, Japan, CK04) according to the manufacturer’s instructions. This was done in growth medium to isolate the direct effect of AOC1 on viability. When testing migration, use Transwell inserts: Cells were seeded in the upper chamber without serum but with different concentrations of recombinant human AOC1 protein, while the lower chamber is the normal growth medium. Use Crystal violet dye staining migrated cells after 24 h.

### 2.6 Immunoprecipitation and Western blotting

When using Co-Immunoprecipitation to test the binding of AOC1 and FBLN1, C2C12 cells were lysed, and lysates were incubated with AOC1 antibody (#A6249, 1:1000, abclonal, Wuhan, China) and IgG antibody as a negative control. Immune complexes were pulled down using protein G agarose beads (#A0812D01, Absin, Shanghai, China). Then doing normal Western blotting procedure to test expression of AOC1 and FBLN1 (#68453-1-lg, 1:1000Proteintech, Hubei, China). When conducting WB, proteins were separated by SDS-PAGE, transferred to PVDF membranes, and probed with antibodies against P53 Monoclonal Antibody (60283-2-Ig, 1:1500, Proteintech, Hubei, China), P16-INK4A Polyclonal antibody (10883-1-AP, 1:1000, Proteintech, Hubei, China), AOC1, Fbln1, FAK (#A2114, 1:1000, abclonal, Wuhan, China), and Phospho-FAK-Y576/Y577 (#AP1547, 1:1000, abclonal, Wuhan, China). Blots were visualized using chemiluminescent substrates.

### 2.7 PCR for skeletal muscles

Total RNA was extracted from the snap-frozen muscle tissues using TRIzol reagent (T9424, Sigma). RNA concentration and purity were determined spectrophotometrically. Complementary DNA (cDNA) was synthesized from 1 µg of total RNA using a reverse transcription kit (PR037A, TaKaRa). Quantitative real-time PCR (qRT-PCR) was performed with the Roche system and specific primers for mouse Aoc1 (Forward: 5′-ACG​GCC​CTG​CTA​TGT​TCA​AG-3'; Reverse: 5′-AGA​GGA​GGT​CAT​ACT​CCG​CTG-3′) and a housekeeping gene Gapdh (Forward: 5′-GGT​TGT​CTC​CTG​CGA​CTT​CA-3'; Reverse: 5′-TGG​TCC​AGG​GTT​TCT​TAC​TCC-3′). The relative mRNA expression level of Aoc1 was calculated using the 2^(–ΔΔCt) method.

### 2.8 Protein extraction and Mass Spectrometry Analysis

Mouse C2C12 myoblasts were cultured in DMEM supplemented with 10% fetal bovine serum (FBS) and 1% penicillin/streptomycin. Cells were treated with 200 pg/mL recombinant human AOC1 protein for 24 h. Cells were lysed using RIPA buffer containing protease inhibitors. Total protein was collected and were incubated with AOC1-specific antibodies overnight at 4 °C, followed by incubation with protein G agarose beads. Bound proteins were eluted, digested with trypsin, and analyzed by LC-MS/MS to identify proteins interacting with AOC1. Identified proteins were analyzed for subcellular localization, and function annotation.

### 2.9 Statistical analysis

Data were analyzed using GraphPad Prism. Significance was determined using T-test or one-way ANOVA with Tukey’s *post hoc* test. *p < 0.05 was considered statistically significant.

## 3 Results

### 3.1 Clinical samples indicate a negative correlation between DAO serum levels and muscle strength

**FIGURE 1:**
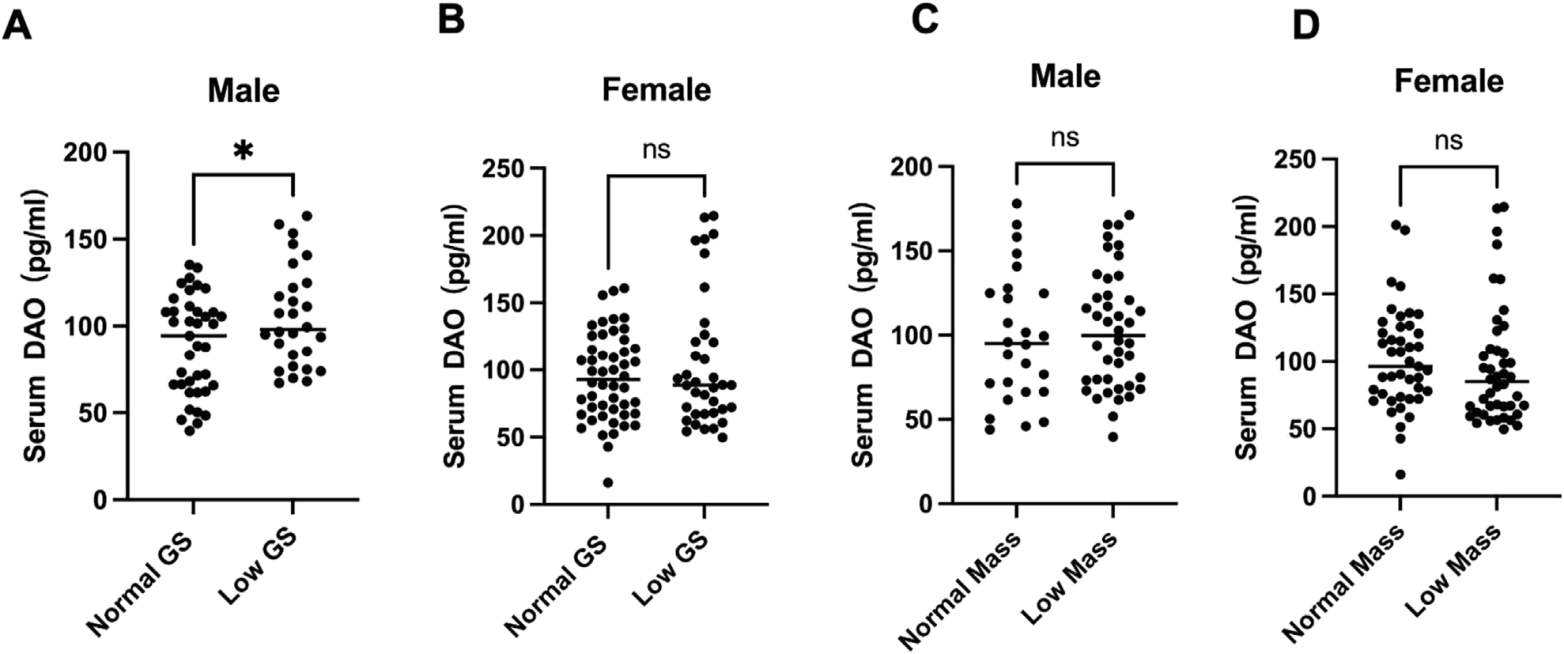
Serum DAO levels in individuals. Serum DAO levels were measured by ELISA. Data are presented as box plots showing the median (central line) and individual data points. **(A)** 68 males over 60 years old serum DAO levels were significantly higher in the low muscle strength group compared to the normal group (*p < 0.05). **(B)** 87 females over 60 years old showed no significant difference (ns) in serum DAO levels between the normal and low muscle strength groups. **(C,D)** Both male and female serum DAO levels showed no significant difference (ns) between the normal and smaller Calf circumference.

While studying the mechanism of osteoporosis and sarcopenia, we enrolled patients admitted to the Orthopedics Department of the First People’s Hospital of Yunnan Province (ethics code KHLL2024-YJ040) from November 2024, after signing informed consent. By December, we included 67 males and 87 females over 60 years old. Handgrip strength was measured three times and averaged. The circumference of each calf at its thickest point was also measured by a tape, and record the larger of the two values. Fasting morning blood was centrifuged to collect serum, and AOC1 levels were determined by ELISA. Using sarcopenia criteria, participants were divided into normal and low grip strength (male <28 kg, female <18 kg) groups. T-test results showed that males in the low grip strength group had lower serum AOC1 than the normal group (Figure 1A), with no significant difference in females (Figure 1B).

### 3.2 Higher DAO content could be detected in weakness skeletal muscles

**FIGURE 2:**
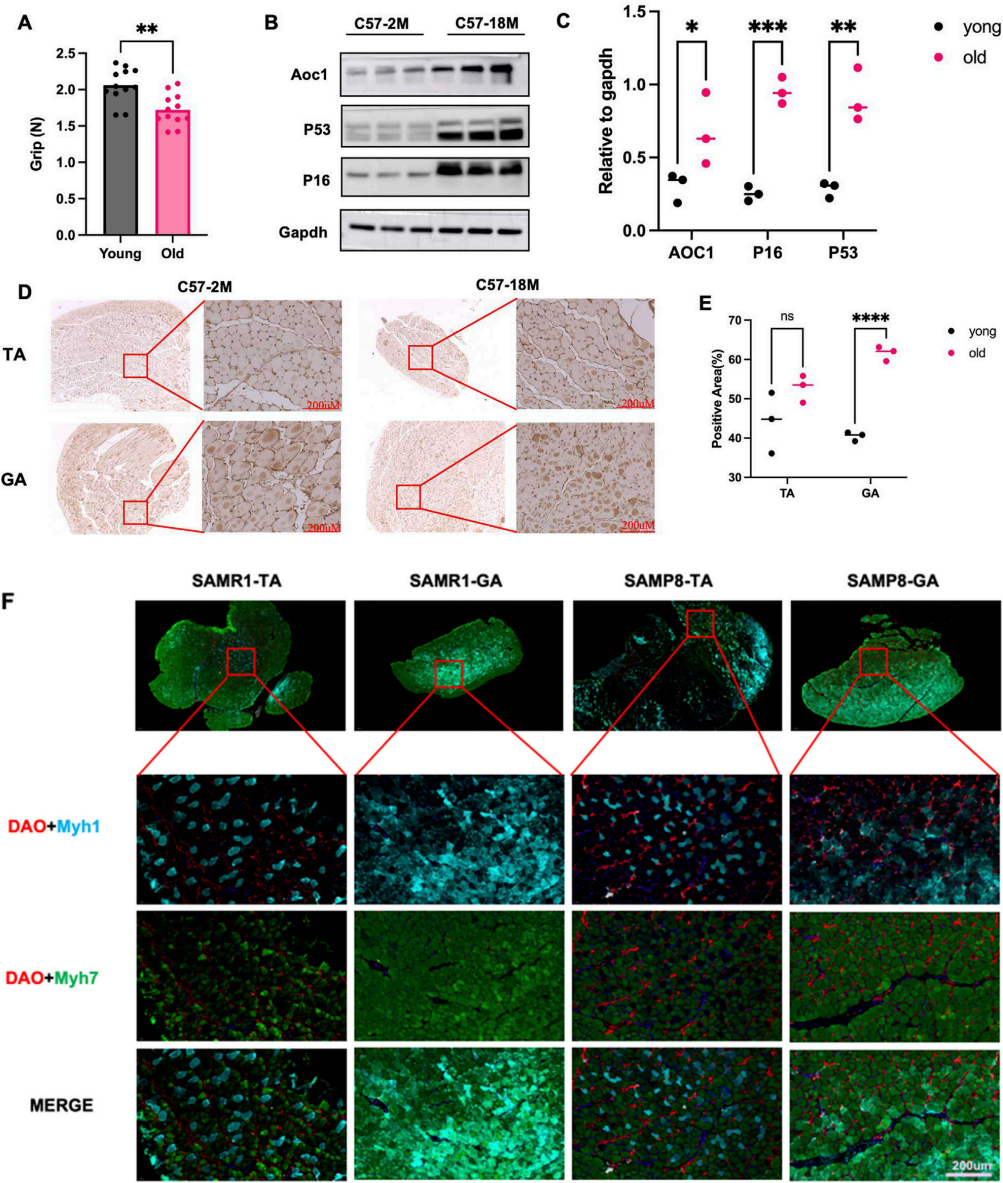
DAO content and muscle strength and muscle fibre types in natural aged mice and rapidly aged mice. **(A)** Grip strength was measured in 4-month-old (young) and 18-month-old (old) male C57 mice using a dynamometer for 4 days. The older group showed significantly reduced grip strength (**p < 0.01). **(B,C)** Western blot analysis of gastrocnemius (GA) muscle lysates showed increased expression of aging markers p53 and p16, along with elevated DAO content in the older group, three replicates were showed in each group. **(D,E)** Immunohistochemistry of GA and tibialis anterior (TA) muscles confirmed higher DAO content (brown) in the older group. Scale bars: 200 μM. Three different area were randomly selected to measure the positive area (%). **(F)** Immunofluorescence staining of tibialis anterior (TA) and gastrocnemius (GA) muscles in 6-month-old male SAMR1 (control) and SAMP8 (accelerated aging) mice. DAO (red) was co-stained with MYH7 (slow muscle fiber marker, green) and MYH1 (fast muscle fiber marker, blue). The merge images show the distribution of muscle fibers. Scale bars: 200 μM.

In 4-month-old and 18-month-old male C57 mice, grip strength was measured with a dynamometer, revealing significantly lower strength in the older group (Figure 2A). In aged group (18-month-old), Gastrocnemius muscle (GA) analysis by Western blotting showed increased expression of aging markers p53 and p16, along with elevated DAO content (Figures 2B,C). Immunohistochemistry on GA and tibialis anterior (TA) muscles also showed higher DAO content (brown) in the aged group (Figures 2C,D).

In the SAMP8 mouse model (a stable accelerated aging strain) and its control SAMR1 strain, gastrocnemius and tibialis anterior muscles from 6-month-old male mice were analyzed. MYH7 (blue-labeled) fast-twitch fibers were markedly reduced in SAMP8 mice, consistent with the pathological feature of fast fiber loss observed in sarcopenia patients (Figure 2F). Concurrently, elevated DAO content was detected alongside this fast fiber reduction. This led us to hypothesize that high DAO content might be harmful for skeletal muscles.

### 3.3 Inflammatory immune micro-environment may also lead to high DAO expression

**FIGURE 3:**
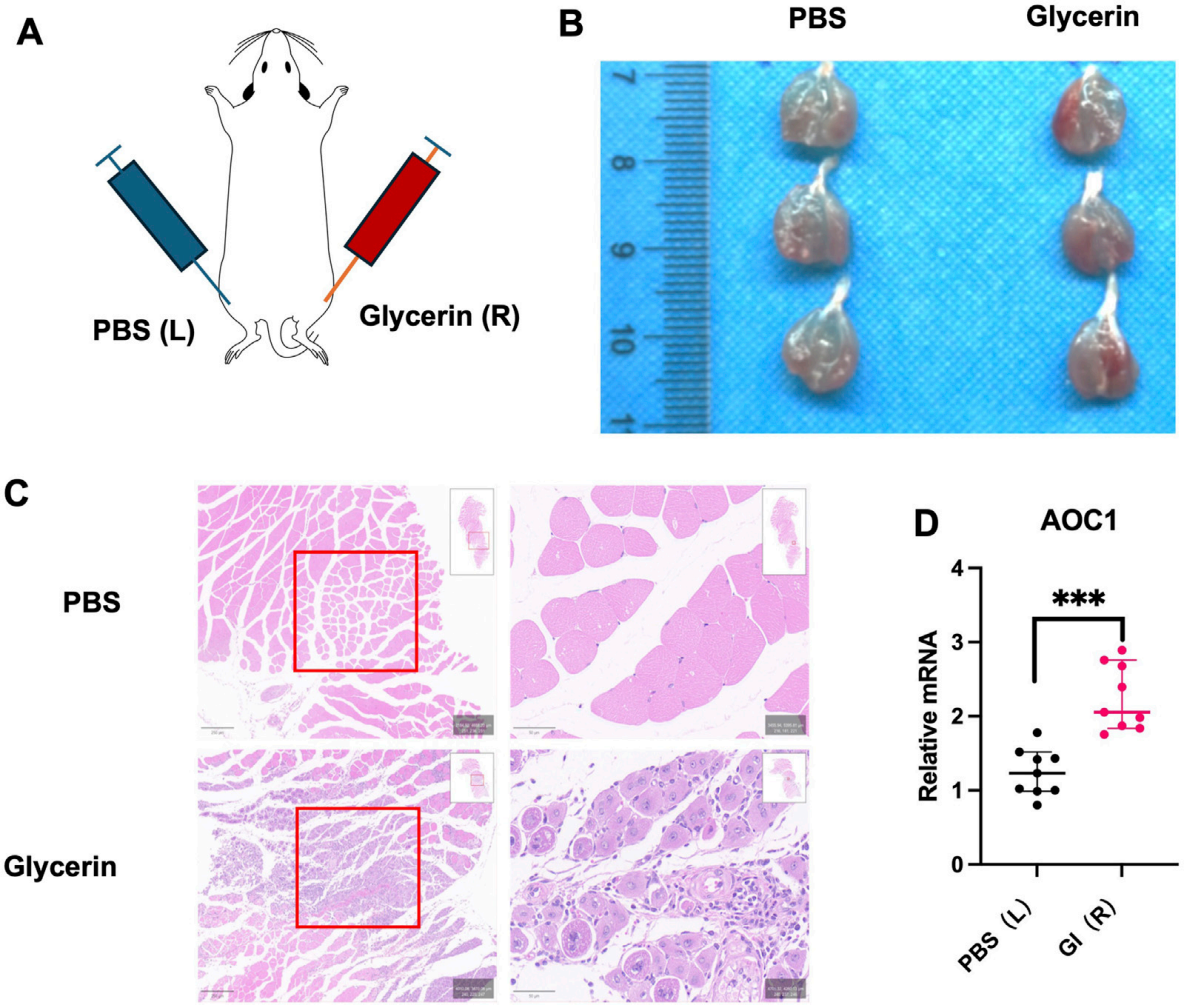
DAO content in acute damaged skeletal muscle model. **(A)** Experimental design for glycerol-induced acute injury in 4-month-old male C57 mice. Glycerol or PBS was injected into the hind limbs. **(B)** Tissue appearance 3 days post-injection showed marked inflammation at glycerol injection sites. **(C)** HE staining of injected tissue revealed histological evidence of inflammation and tissue damage (red boxes). **(D)** qPCR analysis of each group (n = 3) in three times techinal repliates showed increased Aoc1 mRNA levels in glycerol-injected tissues compared to PBS-injected controls (***p < 0.001).

To determine if the DAO can just convey by blood, or can also be produced by located inflammation cells, 4-month-old mice were injected 0.05 mL PBS and glycerol separately (Figure 3A), marked inflammation at glycerol injection sites can be observed by HE staining after 3 days (Figures 3B,C). When taken the whole injured muscle for real-time PCR, higher Aoc1 expression can be detected, considering inflammatory cell can express Aoc1, which also revealing inflammatory cell infiltration.

### 3.4 Overdose recombinant DAO may hinder myoblast migration and fusion by inhibiting the Fbln1/FAK pathway

**FIGURE 4:**
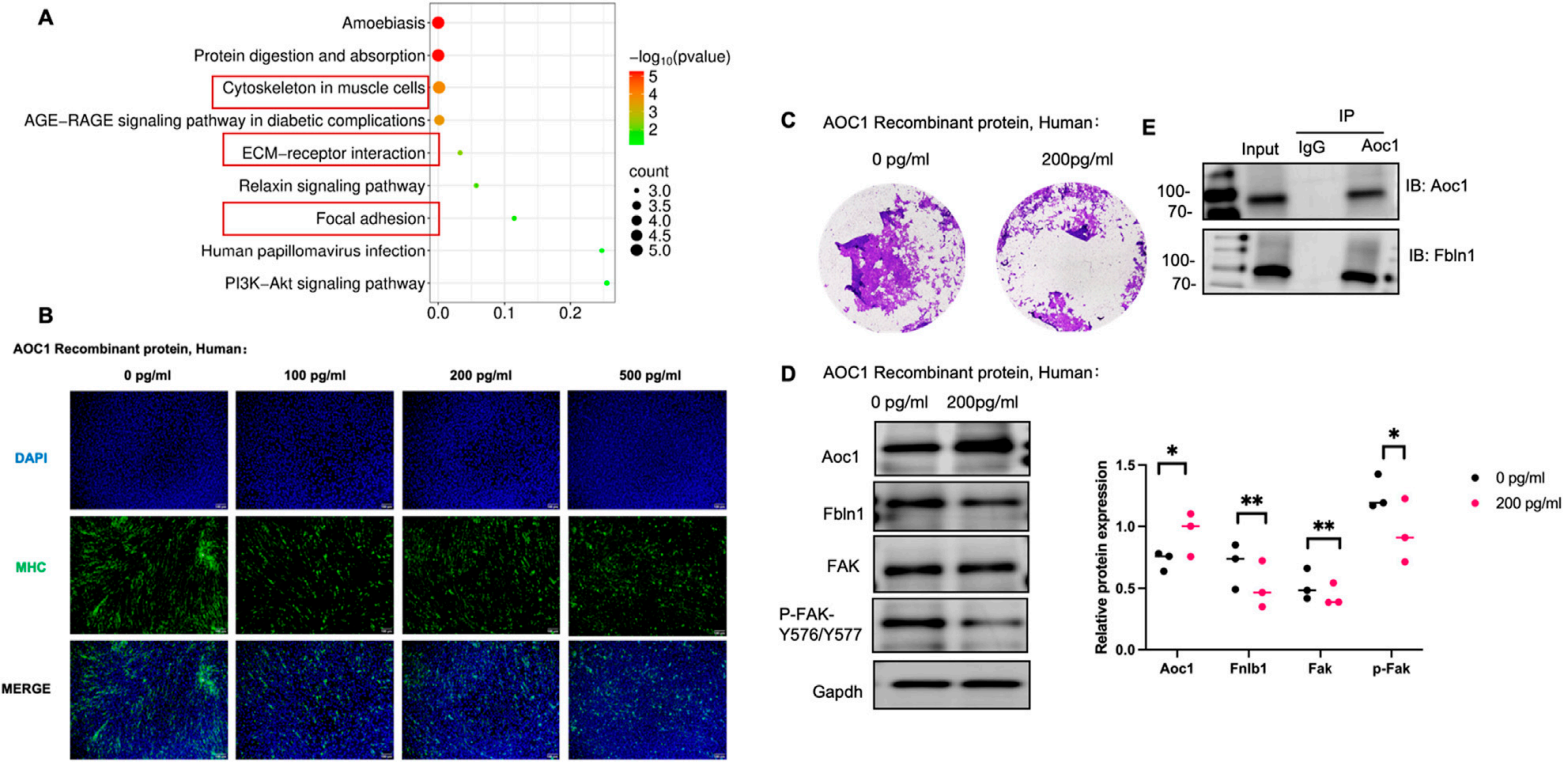
AOC1’s impact on myoblast migration and fusion via the Fbln1/FAK pathway. **(A)** Functional enrichment analysis of proteins binding to AOC1, highlighting enrichment in cytoskeletal and ECM-related proteins. **(B)** Morphological changes in gradient concentration effects of AOC1 on C2C12 cell fusion, showing normal fusion at 0 pg/mL, partial fusion at 100 pg/mL, and disrupted polarity at 200 pg/mL and above. **(C)** Immigration of cell from the upper chamber to down part was showed after AOC1 treatment, visualized by Crystal purple staining. **(D)** Western blot analysis confirming increased AOC1 expression and decreased Fbln1 and FAK phosphorylation (P-FAK -Y576/Y577) at higher AOC1 addtion. **(E)** Co-immunoprecipitation (IP) confirming AOC1’s interaction with Fbln1.

To further explore the effect of increasing serum DAO level on muscle cells, revealing the receptor on muscle cells, adding 200 pg/mL recombinant human DAO protein to cultured C2C12 cells and incubating for 24 h, proteins were collected and analyzed via mass spectrometry to identify those binding to DAO protein. Subcellular localization analysis showed that exogenous DAO binds to many cytoskeletal proteins, potentially involing cell migration and adhesion (Figure 4A). Further experiments with gradient concentrations of AOC1 revealed normal fusion in controls, less fusion but polar arrangement at 100 pg/mL, and disrupted polarity and no fusion at 200 pg/mL and 500 pg/mL (Figure 4B). Transwell assays confirmed reduced migration in the 200 pg/mL group (Figure 4C). Mass spectrometry highlighted high abundance of Fbln1 and Fbln2. Fbln1 (Fibulin-1) is an extracellular matrix (ECM) glycoprotein-encoding gene that plays a critical role in regulating cell adhesion (Ji et al., 2024), migration, and tissue remodeling. In myoblasts, Fbln1 promotes cell migration and fusion by interacting with integrin receptors to activate downstream FAK (Focal Adhesion Kinase) signaling (Li et al., 2023), which enhances cytoskeletal reorganization and mechanotransduction, facilitating myoblast alignment and fusion into multinucleated myotubes. Considering of this, we examined the expression of Fbln1 after the addition of AOC1 and its influence on downstream FAK signaling pathways (Figure 4D), and preliminarily verified the combination of AOC1 and Fbln1 (Figure 4E).

**FIGURE 5:**
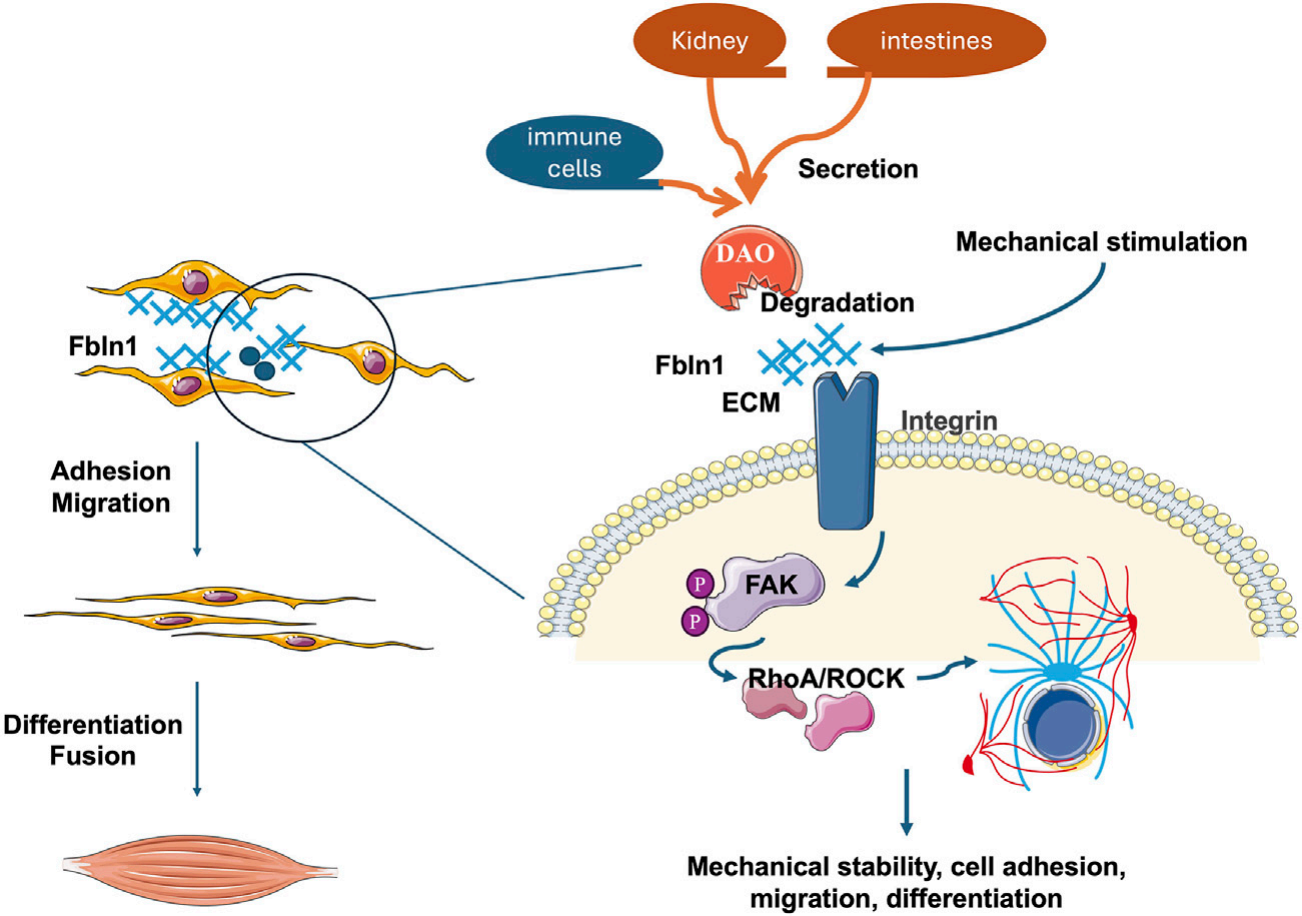
The illustration diagram showing the role of DAO in muscle cell behavior and signaling pathways. Mechanical stimulation activates Fbln1 in the extracellular matrix (ECM), which influences cell adhesion and migration through integrin receptors on the cell membrane. The binding of Fbln1 to integrins triggers the activation of Focal Adhesion Kinase (FAK). Once activated, FAK phosphorylates and recruits downstream effectors like RhoA/ROCK, leading to cytoskeletal rearrangements and mechanical signal transduction. These processes are crucial for maintaining mechanical stability, cell adhesion, migration, and differentiation, ultimately affecting muscle cell fusion and function. But when DAO combined and inhibite the function of Fbln1, the whole mechanical conduction pathway is disturbed.

To integrate our findings into a cohesive model, we propose a working mechanism whereby excessive DAO impairs skeletal muscle regeneration and function by sabotaging the critical Fbln1/FAK mechanotransduction pathway (as summarized in Figure 5). Under physiological conditions, mechanical stimulation promotes the engagement of the extracellular matrix (ECM) protein Fbln1 with integrin receptors on the myoblast membrane. This interaction initiates the phosphorylation of Focal Adhesion Kinase (FAK) at residues Y576/Y577, which in turn activates downstream effectors such as RhoA/ROCK to drive cytoskeletal reorganization. This process is essential for myoblast migration, alignment, and ultimate fusion into multinucleated myotubes. Our data demonstrate that elevated DAO, either from circulation or local inflammation, binds directly to Fbln1. This binding likely sequesters Fbln1 or alters its conformation, thereby uncoupling mechanical signals from integrin-mediated FAK activation. The consequent attenuation of FAK phosphorylation and disruption of the cytoskeletal dynamics lead to aberrant cell polarity, inhibited migration, and failed fusion. This model positions DAO as a pivotal disruptor of metabolic-biomechanical crosstalk, providing a mechanistic explanation for the observed decline in muscle strength associated with high DAO levels.

## 4 Discussion

The present sequence data unveils AOC1 might be a novel regulator of muscle strength, yet lack of research evidance to make sure, one important question is the content of AOC1 in skeletal muscles is super low, revealing the possibility of organ interaction. Our findings across clinical cohorts and preclinical models provide compelling evidence that AOC1 maybe a pivotal mediator of metabolic-biomechanical crosstalk in skeletal muscle, which modulates muscle function through disrupting mechanotransduction pathways critical for myoblast fusion.

The clinical observation of inverse correlation between serum AOC1 levels and grip strength (Figure 1A) aligns with preclinical data showing elevated DAO content in aged and injured muscles (Figures 2, 3). Notably, this association was sex-specific, with males exhibiting significant reductions in grip strength alongside higher DAO content, while females showed no such trend (Figure 1B). This discrepancy may reflect hormonal influences or sex-dependent differences in AOC1 metabolism, warranting further investigation.

The age- and injury-associated upregulation of AOC1 coincided with selective depletion of fast-twitch fibers (MYH7^+^), as demonstrated in both SAMP8 mice and AOC1-overexpressing models (Figure 2F). Fast fibers, which rely on glycolytic metabolism, are particularly vulnerable to sarcopenia (Blemker et al., 2024). We propose that AOC1 exacerbates this vulnerability by disrupting Wnt signaling due to the appearance of Wnt receptor LRP5, PTK7 in Mass Spectrometry Analysis results (Supplementary Table 1). The specification and maintenance of fast-twitch (glycolytic, Type II) muscle fibers are critically regulated by Wnt/β-catenin signaling (Dowling et al., 2023), a pathway that bridges mechanical stimuli to transcriptional reprogramming. High-intensity resistance training, such as weightlifting, generates intermittent mechanical tension, which activates Wnt ligands in skeletal muscle. These ligands bind to Frizzled receptors, stabilizing β-catenin by inhibiting its proteasomal degradation. Accumulated β-catenin translocates to the nucleus, where it partners with TCF/LEF transcription factors to drive the expression of fast-twitch-specific genes, including Myosin Heavy Chain isoforms (MyHC IIa/IIx) (Cisternas et al., 2014). Thus, this might be a hint that AOC1 can competitively bind to receptors like LRP5 or PTK7, inhibit the Wnt/β-catenin signaling, reduce the number of fast fibers.

**TABLE 1:** Baseline characteristics of study participants stratified by grip strength.

Characteristic	Normal grip strength (n = 89)	Low grip strength (n = 66)	P-value
Demographics			
Male, n (%)	39 (43.82%)	29 (43.94%)	
Age (years)	70.15 ± 6.548	72.72 ± 7.468	0.1364
Anthropometrics			
Calf circumference (cm)	34.21 ± 3.420	30.98 ± 3.600	<0.001
Muscle strength			
Grip strength (kg)	35.04 ± 6.301	19.49 ± 6.500	<0.001

Our mechanistic studies reveal that DAO may directly interferes with Fbln1-dependent FAK activation, a master regulator of myoblast migration and fusion (Figures 4D,E). Fbln1, an ECM glycoprotein, typically binds integrins to initiate FAK phosphorylation (P-FAK-Y576/Y577), triggering cytoskeletal remodeling via RhoA/ROCK(Holland et al., 2024). AOC1’s interaction with Fbln1 (Figure 4E) likely sequesters Fbln1 from integrins, thereby uncoupling mechanical forces from downstream signaling. This disruption manifests as aberrant cell polarity (Figure 4B) and reduced migration (Figure 4C), ultimately impairing myotube formation—a process requiring precise spatial coordination of fusion-competent myoblasts.

All in all, this work positions AOC1 at the nexus of muscle metabolism and biomechanics (Figure 5), offering a unifying framework to explain age-related strength loss under whole body state. The results revealing except liver, kidney, intestines, and immue cells secretory proteins may also involved in muscle regulation. By hijacking Fbln1/FAK signaling, DAO not only rewires muscle energy metabolism but also erodes the mechanical infrastructure essential for force production—a dual assault that underscores its potential as a therapeutic target for sarcopenia and related myopathies.

## Data Availability

The datasets presented in this study can be found in online repositories. The names of the repository/repositories and accession number(s) can be found in the article/Supplementary Material.
